# Raltitrexed treatment promotes systemic inflammatory reaction in patients with colorectal carcinoma

**DOI:** 10.1038/sj.bjc.6600520

**Published:** 2002-09-09

**Authors:** P Österlund, A Orpana, I Elomaa, H Repo, H Joensuu

**Affiliations:** Department of Oncology, Helsinki University Central Hospital, Haartmaninkatu 4, FIN-00029 Helsinki, Finland; Department of Clinical Chemistry, Helsinki University Central Hospital, Haartmaninkatu 4, FIN-00029 Helsinki, Finland; Department of Internal Medicine, Helsinki University Central Hospital, Haartmaninkatu 4, FIN-00029 Helsinki, Finland; Department of Bacteriology and Immunology, The Haartman Institute, Helsinki University Finland

**Keywords:** colorectal cancer, raltitrexed, carmofur, 5-fluorouracil, cytokine, systemic inflammation

## Abstract

We studied longitudinally inflammatory reactions and serum C-reactive protein (S-CRP) levels in 52 colorectal cancer patients treated with a median of six 3-weekly cycles of raltitrexed 1.5–3.0 mg m^−2^ combined with oral carmofur (1-hexylcarbomoyl-5-fluorouracil) 300–400 mg m^−2^ on cycle days 2–14. Thirty-nine (75%) of these patients had fever on days 2 to 9 after receiving raltitrexed, 49 (94%) had fatigue Gr.⩾1, and 49 (94%) elevated S-CRP without a documented infection. The systemic inflammatory composite score (consists of body temperature, fatigue, S-CRP, interleukin-6 (S-IL-6), S-IL-8, and tumour necrosis factor-α (S-TNFα) levels) was calculated in a cross-sectional one-cycle study involving 60 colorectal cancer patients treated with single-agent raltitrexed, raltitrexed and carmofur, or 5-fluorouracil-based chemotherapy (*n*=20 in each group). The median S-CRP, S-IL-6, and S-TNFα levels were higher 7 days after giving raltitrexed (57 *vs* 23 mg l^−1^, 64 *vs* 10 ng l^−1^, and 11 *vs* 10 ng l^−1^, respectively) or raltitrexed+carmofur (142 *vs* 10 mg l^−1^, 64 *vs* 10 ng l^−1^, and 16 *vs* 9 ng l^−1^, respectively) than at baseline (*P*<0.01 for each comparison), but not when 5-fluorouracil-based regimens were administered. These findings suggest that colorectal cancer patients treated with raltitrexed may develop drug-related systemic inflammation, which may be difficult to discriminate from infection.

*British Journal of Cancer* (2002) **21**, 591–599. doi:10.1038/sj.bjc.6600520 www.bjcancer.com

© 2002 Cancer Research UK

## 

Raltitrexed (Tomudex®), designed as a specific thymidylate synthase inhibitor, has shown comparable efficacy to the combination of bolus 5-fluorouracil (5-FU) and leucovorin in the treatment of metastatic colorectal cancer (Cocconi *et al*, 1998; Cunningham *et al*, 1996; Pazdur and Vincent, 1997). However, the toxicity profile is dependent on the regimen (Cunningham *et al*, 1996; Garcia-Vargas *et al*, 1999; Pazdur and Vincent, 1997), and, in general, raltitrexed causes less leukopenia and mucosal injury than 5-FU combined with leucovorin, but more fatigue, fever and transient increases in the hepatic transaminase levels.

Raltitrexed has been extensively investigated as a single drug, and its use in combination therapies is currently under clinical evaluation. We have recently studied the combination of raltitrexed and carmofur (1-hexylcarbomoyl-5-FU), an orally administered 5-FU derivate that is metabolised to FdUMP and acts as intravenous 5-FU (Miyauchi *et al*, 2000), as the first-line treatment of colorectal carcinoma. This combination has a promising 50% response rate in first line when raltitrexed is given 1.5 to 3.0 mg m^−2^ on day 1 and carmofur 300 to 400 mg m^−2^ on days 2 to 14 of a 3-week cycle (Osterlund *et al*, 2001). Many of the patients treated with this combination or with single-agent raltitrexed presented with high spiking fever and fatigue within 72 h of raltitrexed infusion, followed by an increase in the serum C-reactive protein (CRP) level, while the blood neutrophil counts remained virtually unaltered. Despite extensive clinical and imaging examinations, presence of infection could not be verified in these patients. Recovery was invariably complete within one week irrespective of the type of treatment given (broad spectrum antibiotics or oral ofloxacin), or whether symptomatic care only without antibiotics was given. Similar symptoms typically recurred following further raltitrexed infusions in the same patients, which also suggested the presence of non-infectious, systemic inflammation.

The triad of fever, fatigue, and an increased serum CRP level is typical of systemic inflammation. The most common cause of systemic inflammation is infection (Anonymous, 1992; Bone, 1996), but since no evidence for recurring infections was obtained in our patients and antimicrobial therapy appeared to have no effect on the outcome, we assumed that the treatment itself might trigger a systemic inflammatory reaction in these patients. To evaluate this hypothesis we carried out two prospective studies, one longitudinal and one cross-sectional study, which are reported here. In the longitudinal study the frequency of fever, fatigue, and an increased serum CRP level were investigated in patients who were treated with the combination of raltitrexed and carmofur for metastatic colorectal cancer, and the responsiveness of the symptoms to dexamethasone. In the one-cycle cross-sectional study, the severity of systemic inflammation during one cycle of treatment was compared between patients treated either with single-agent raltitrexed, the combination of raltitrexed and carmofur, or a 5-FU-based regimen. In the cross-sectional study we measured body temperature, the degree of fatigue, and the serum levels of CRP, TNF-α, IL-6, and IL-8 as markers of systemic inflammation. Based on these analyses we calculated the systemic inflammation composite score (SICS) to evaluate the severity of inflammation in individual patients (Takala *et al*, 1999).

## PATIENTS AND METHODS

### Patients

The protocols of the longitudinal and cross-sectional studies were approved by the local Ethical Review Board and the National Agency for Medicines, Helsinki, Finland, and an informed consent was required from all patients. Both studies were open and non-randomised. Patients were eligible for inclusion if they were older than 18 (and younger than 75 in the longitudinal study); had histologically confirmed metastatic or locally advanced inoperable colorectal cancer; the World Health Organisation (WHO) performance status ⩽2; and had adequate bone marrow, renal, and liver functions at initiation of treatment. Exclusion criteria included the presence of other invasive cancer, with the exception of adequately treated carcinoma *in situ* of the cervix and non-melanoma skin cancer; metabolic, neurological, or psychiatric disease that was incompatible with chemotherapy; a history of serious thromboembolic event currently under treatment; pregnancy, lactation, or absence of adequate contraception in fertile patients; and in the cross-sectional study infection between days 0 and 9 of the treatment cycle.

The longitudinal study comprised of 52 patients treated with 240 cycles of raltitrexed and carmofur. The study included all patients who participated in a single institution phase I/II study investigating the dose and efficacy of the combination of raltitrexed and carmofur at the Helsinki University Central Hospital between March 1998 and November 1999 as reported earlier (Osterlund *et al*, 2000, 2001). The patient characteristics are shown in Table 1

**Table 1:**
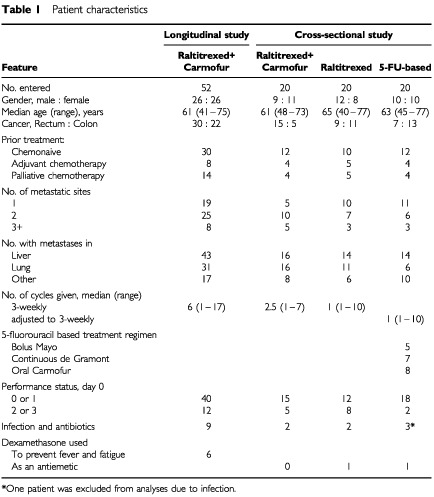
Patient characteristics

.

The cross-sectional one-cycle study comprised of 60 consecutive patients with metastatic colorectal cancer treated at the Helsinki University Central Hospital (Table 1). The patients were treated with one of three different chemotherapies. Twenty patients were treated with raltitrexed and carmofur (they also participated in the longitudinal study); 20 patients received raltitrexed as a single-agent therapy; and 20 patients received 5-FU in combination with leucovorin (either the Mayo 5-day bolus injection regimen or the de Gramont regimen were used), or single-agent carmofur. The three treatment arms were balanced with respect of gender, age, disease sites, and prior treatments (Table 1).

### Chemotherapy regimens

Raltitrexed 3.0 mg m^−2^ was given as a 15 to 30 min infusion 3-weekly, the starting day of the next cycle was day 22. When given in combination with carmofur, raltitrexed 1.5 to 3.0 mg m^−2^ was administered on the cycle day 1 and carmofur 300 to 400 mg m^−2^ orally divided in three daily doses on cycle days 2–14, followed by a week of rest (Osterlund *et al*, 2001). The ‘Mayo’ regimen was administered as bolus injections of leucovorin 20 mg m^−2^ and 5-fluorouracil 425 mg m^−2^ on days 1 to 5 of the cycle, repeated every four weeks. The ‘simplified de Gramont’ regimen was given every 2 weeks with leucovorin 400 mg m^−2^ as a 2-h infusion followed by 5-fluorouracil 400 mg m^−2^ given as a bolus, followed by a continuous infusion of 5-fluorouracil 3.0 to 3.6 g m^−2^ 48 h using a portable pump. Single-agent carmofur was administered as 3-week cycles, where carmofur 300 mg m^−2^, divided in three daily doses, was first given orally for 14 days, followed by 1 week of rest. The number of cycles was not limited in the longitudinal study, and the combination of raltitrexed and carmofur was given until disease progression or dose-limiting toxicity was encountered.

### Clinical evaluation

At study entry physical examination was performed, the performance status evaluated, and the European Organization for Research and Treatment of Cancer (EORTC) quality of life questionnaire (QLQ) C-30 forms were filled. At the end of each cycle physical examination, the performance status, and treatment toxicities using the National Cancer Institute of Canada toxicity Criteria were assessed. In the longitudinal study body temperature was measured at least daily during the first days of the cycle and whenever fever was suspected. In the cross-sectional study, body temperature was measured at least every morning, QLQ was assessed once weekly, and patients kept a diary of side-effects and of general well-being. Response to treatment was assessed according to the World Health Organization (WHO).

Special attention was paid to infection diagnostics of febrile patients. In addition to careful clinical examinations, urine and blood samples were collected repeatedly for bacterial and fungal culture, antigen tests, and microbial serology. Conventional radiological imaging, ultrasound, or computed tomography were used to find infection foci.

In the longitudinal study, all infectious episodes started on cycle days 10 to14 at the time of the leukocyte nadir, or later than this during the treatment cycle. Four patients were diagnosed with infection (urinary tract infection, *n*=1; biliary stent sepsis, *n*=1; pneumonia, *n*=1; erysipelas, *n*=1). In addition, five patients had a suspected infection with concurrent Gr. 3 or 4 neutropenia and diarrhoea.

In the cross-sectional study, seven patients developed infection or suspected infection during the study cycle and were treated with antibiotics. Pneumonia occurred in one patient, enteritis of unknown origin in one, urinary tract infection in three, and urinary tract infection was suspected in two patients. One of the urinary tract infections was a septic urinary catheter infection and started already on day 4 of the cycle, whereas the other infectious episodes began on cycle days 11 to 21, i.e. after the blood samples for the laboratory tests for the presence of systemic inflammation had been collected.

### Laboratory tests

Blood haemoglobin, the white blood cell and leukocyte differential counts, the platelet count, and the erythrocyte sedimentation rate (ESR) were measured, as well as serum CRP, alkaline phosphatase, alanine aminotransferase (SGPT), aspartate aminotransferase (SGOT), bilirubin, creatinine, Na, K, and calcium levels at the study entry prior to the first chemotherapy infusion. In the longitudinal study these tests were repeated on day 7 or 8 of the cycle, and immediately prior to the next cycle. The blood tests were repeated every second day if grade IV cytopenia was present. In the cross-sectional study, these tests were done thrice during each chemotherapy cycle (on cycle day 6 to 8 and 12 to 15, and before starting the next cycle).

Immulite (Diagnostic Products, Los Angeles, CA, USA) chemiluminescence analysis was used to measure the serum levels of TNF-α (the detection limit, 1.7 ng l^−1^), IL-6, and IL-8 (the detection limit, 5 ng l^−1^ for each) as described previously (Takala *et al*, 2000). The cytokine levels were determined in the cross-sectional study at the study entry, and once on days 6 to 8, 12 to 15, and at the end of the cycle. The serum cytokines levels could be measured on the four planned times in 59 patients, and three times in one case. An insufficient amount of plasma was obtained in one case on the day 7 sample, and only two of the cytokines could be analysed.

### Evaluation of systemic inflammation

To evaluate the severity of systemic inflammation we determined the systemic inflammation composite score (SICS) (Takala *et al*, 1999). To obtain the SICS, a subscore (grade) was first assigned to the body temperature (graded from 0 to 4), fatigue (from 0 to 3), and the serum levels of CRP, TNF-α, IL-6, IL-8 (each graded from 0 to 4, Table 2

**Table 2:**
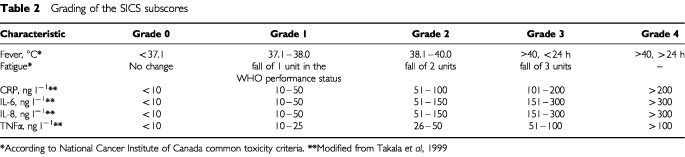
Grading of the SICS subscores

). The maximum obtainable SICS was thus 5×4 + 1×3, i.e. 23, where a high score denotes the presence of severe systemic inflammation. The severity of systemic inflammation was assigned for the 60 patients who participated in the cross-sectional study four times; once before the cycle, and on cycle days 6 to 8, 12 to 15, and at the end of the cycle.

### Study endpoints and statistical methods

In the longitudinal study, nine treatment cycles were excluded from analysis due to the presence of a diagnosed infection and subsequent antimicrobial therapy. Six patients in the longitudinal study were treated with intravenous and/or oral dexamethasone during a total of 17 treatment cycles to diminish symptoms related to systemic inflammation (see below). Data from these 17 cycles were also excluded from the main analyses. Repeated measures ANOVA statistics was used to examine changes in variables over time.

In the cross-sectional study the SICS score could not be calculated in three patients; in two patients due to a missing value (one patient in the 5-FU group and one in the raltitrexed group), and in one case due to the presence of infection (the 5-FU group). The Mann–Whitney *U*-test or the Kruskall–Wallis test were used to compare groups for the difference between the day 0 (the baseline value before staring treatment) and day 7 (Δday 0 to 7) values, and the Wilcoxon signed rank test for paired comparisons. The SICS analysis was done with and without the six patients who had an infectious episode and the two patients who received a single dose of 10 mg intravenous dexamethasone prior to chemotherapy as an antiemetic (Table 1). All statistical significance tests are two-tailed at the 5% level. Bonferroni correction for multiple comparisons was used when appropriate.

## RESULTS

### The longitudinal study

Body temperature, fatigue, and serum CRP during raltitrexed and carmofur treatment. Fever and/or fatigue occurred during 161 (75%) of the 214 cycles given to 52 patients (fever >37.0°C, *n*=39; >38.0°C, *n*=24). Fatigue grade ⩾1 was present in 49 (94%) patients, and other commonly recorded symptoms included anorexia with slight nausea, and flu-like symptoms with headache and myalgia. Serum CRP level increased more than 10 mg l^−1^ in 49 (94%) and the ESR more than 10 mm h^−1^ in 45 (87%) patients.

**Figure 1:**
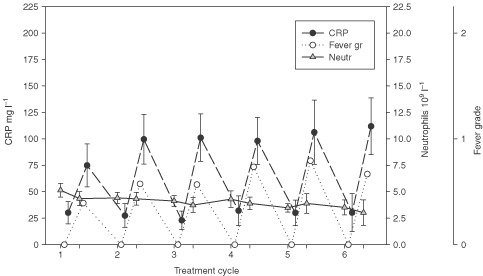
The serum C-reactive protein (CRP) level, presence of fever (graded as in Table 2), and the blood neutrophil count in the longitudinal study. The mean and 95%CI of the 231 cycles given to 52 patients are indicated by bars.

Body temperature followed a characteristic pattern, peaking 2 to 4 days after raltitrexed administration, and resolving usually within 4 days thereafter (range, 1 to 7 days). A rise in body temperature was accompanied with a rise in the serum CRP level and the blood ESR, which reached the maximum values on about cycle days 7 to 8, and then returned to the precycle levels by the first day of the next cycle. The frequency of fever (*P*=0.0085) and elevation of serum CRP (*P*=0.0014) increased over the first three cycles of carmofur plus raltitrexed treatment, whereas no marked change took place in the mean blood neutrophil levels (*P*=0.25, Figure 1

). No significant change in the median baseline (precycle) serum CRP levels was found during the first six cycles of carmofur and raltitrexed treatment (*P*=0.27). One of the four patients treated at raltitrexed dose levels 1.5 or 2.0 mg m^−2^, and eight of the 16 patients treated at dose levels 2.5 or 3.0 mg m^−2^ developed dose limiting symptoms or signs suggestive of systemic inflammatory reaction (*P*=0.59, Fisher's exact test).

#### Effect of dexamethasone

Six patients treated with the combination of carmofur and raltitrexed and who suffered from recurring post-infusion fever and fatigue, were given dexamethasone in an attempt to prevent the post-infusion systemic inflammatory reaction. A single dose of 10 mg intravenous dexamethasone given at the time of raltitrexed infusion failed to prevent the symptoms, but when 4.5 mg of dexamethasone t.i.d. was added to the regimen and when this dose was tapered down over 10 days, neither fever, fatigue, the flu-like symptoms nor elevations of CRP and ESR recurred during the subsequent chemotherapy cycles in all patients. Interestingly, five of the six patients evaluable for treatment response progressed after starting dexamethasone for prevention of the symptoms.

### The cross-sectional study

#### Body temperature, fatigue, serum CRP level and the blood neutrophil counts

**Figure 2:**
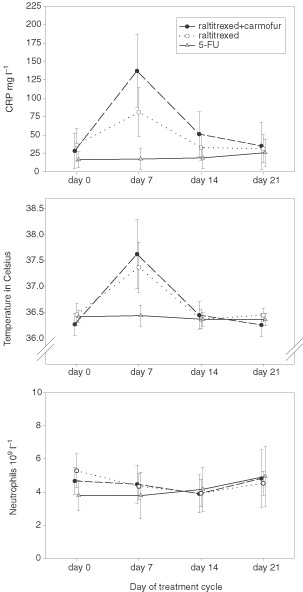
The serum CRP level, body temperature, and the blood neutrophil count as assessed during one cycle of chemotherapy in 59 patients treated with three different chemotherapies (the cross-sectional study).

The changes in the serum CRP level, body temperature, and the blood neutrophil counts during one cycle of chemotherapy consisting either of raltitrexed plus carmofur, single-agent raltitrexed, or a 5-FU based regimen are shown in Figure 2

(*n*=59). The post-infusion elevations of serum CRP and body temperature were highly significant as compared to the precycle values (*P*=0.0001 and <0.0001, respectively). The serum CRP level and the body temperature peaked on the cycle day 7 in patients treated with either carmofur+raltitrexed or single-agent raltitrexed (repeated measures analysis of variance *P*=0.021 and 0.084, respectively), but no such elevations were observed in patients who received 5-FU based chemotherapy. Grade 3 or 4 neutropenia was found only in four (7%) of the 59 patients during the chemotherapy cycle with no statistically significant difference between the treatment groups (*P*=0.89), and no significant changes in the blood neutrophil counts took place during the cycle (*P*=0.53, Figure 2).

**Figure 3:**
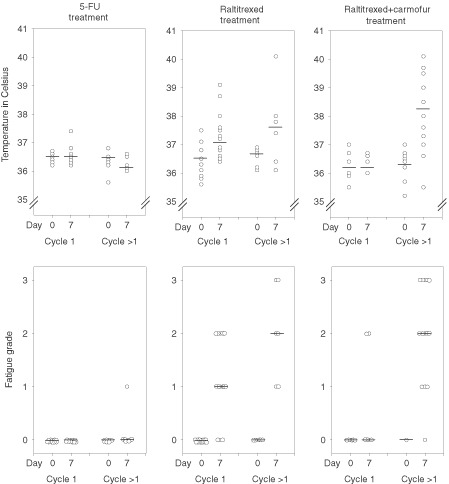
Body temperature and the fatigue grade of 59 patients who participated in the cross-sectional study. The results are shown split by the type of treatment given and the chemotherapy cycle assessed (cycle 1, evaluation was carried out during the first chemotherapy cycle; cycle >1, evaluation was done during one of the later cycles). The individual patient values (open circles) and the medians (horizontal lines) are indicated. Day 0 refers to the baseline (precycle) value, and day 7 is the 7^th^ day of the chemotherapy cycle.

Elevated body temperature (range, from 37.0 to 40.1°C) occurred more frequently in patients who received single-agent raltitrexed or raltitrexed plus carmofur than in patients treated with 5-FU based regimens (*n*=11 *vs* 11 *vs* 1, *P*=0.0091). The median body temperature was higher on cycle day 7 (37.3°C, 37.5°C and 36.4°C, respectively) as compared with the baseline (36.3 to 36.5°C) in patients treated with the raltitrexed-containing regimens (*P*=0.0001). Patients with fever had also fatigue and usually flu-like symptoms (anorexia, myalgia, and headache). The fatigue grade was lower in patients treated with the 5-FU based regimens than in those who received raltitrexed-based therapy (median, 0 *vs* 1 *vs* 1, *P*<0.0001), and the increase in fatigue was also greater in the raltitrexed arms than in the 5-FU based therapy arm based on the quality-of-life questionnaires. The treating physician-estimated degree of fatigue and the patient-reported fatigue based on the QLQs showed strong correlation (Spearman rank correlation coefficient −0.632, *P*<0.001). As in the longitudinal study, the changes in the body temperature and fatigue were smaller during the first chemotherapy cycle as compared to the later cycles (Figure 3

).

#### Markers of systemic inflammation

**Figure 4:**
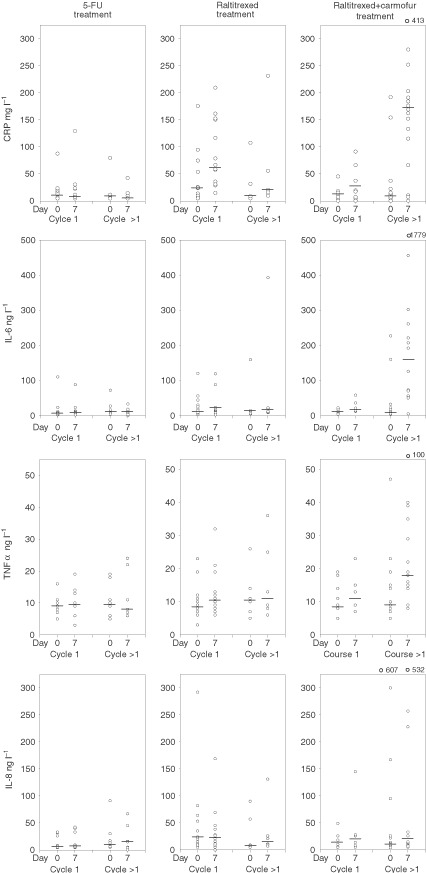
Serum levels of CRP, IL-6, IL-8, and TNFα of 59 patients in the cross-sectional study. The symbols and cycles are as in Figure 3.

The median serum CRP, IL-6, IL-8, and TNFα levels were higher 7 days after starting raltitrexed and carmofur therapy as compared to the precycle levels (142 *vs* 10 mg l^−1^ 64 *vs* 10 ng l^−1^, 21 *vs* 11 ng l^−1^, and 16 *vs* 9 ng l^−1^, respectively, *P*<0.001 for each comparison). Similarly, the median serum levels of CRP, IL-6, and TNFα were significantly higher on the cycle day 7 as compared with the precycle levels after commencing single-agent raltitrexed (57 *vs* 23 mg l^−1^, 64 *vs* 10 ng l^−1^, and 11 *vs* 10 ng l^−1^, respectively, *P*<0.01 for each comparison), but no similar effect was found for IL-8 (22 *vs* 21 ng l^−1^). In contrast, patients treated with the 5-FU-based regimens had unchanged serum levels of CRP (6 *vs* 7 mg l^−1^), IL-6 (9 *vs* 7 ng l^−1^), TNFα (9 *vs* 9 ng l^−1^), and IL-8 (7 *vs* 7 ng l^−1^). The increases in the serum cytokine levels were more marked in patients who had received more than one cycle of raltitrexed-containing therapies than in those who were assessed during the first chemotherapy cycle (Figure 4

).

#### The systemic inflammatory composite score (SICS)

**Figure 5:**
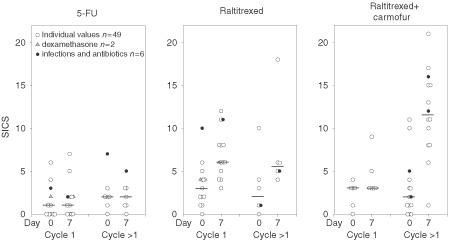
The systemic inflammatory composite score (SICS) of 57 patients who participated in the cross-sectional-study. The symbols and cycles are as in Figure 3.

When the systemic inflammatory composite score was calculated both at the baseline (before giving chemotherapy infusion) and on day 7 of the chemotherapy cycle, the score increased markedly in patients who received raltitrexed (*P*<0.0001). The median cycle day 7 score was higher than the precycle score in patients treated with either raltitrexed and carmofur (9.5 *vs* 2.5, *P*=0.0003) or single-agent raltitrexed (6 *vs* 3, *P*=0.0004), whereas in patients given 5-FU based regimens the score remained unaltered (1.5 *vs* 1.5). The increase in the score was smaller in patients who were assessed during the first chemotherapy cycle as compared with those assessed during the later cycles (*P*=0.027, Figure 5

). Raltitrexed-treated patients had significantly higher cycle day 7 scores irrespective of whether only the patients assessed during the first cycle or those assessed during the later cycles were included in the analysis. The results remained essentially unchanged when patients with a documented infection during the cycle (*n*=6) and those who received dexamethasone as an antiemetic (*n*=2) were excluded (in the raltitrexed plus carmofur group the median day 7 SICS was 8.5 *vs* 2.5 at baseline, *P*=0.0006; in the single-agent raltitrexed group 6 *vs* 2.5, *P*=0.0007; and in the 5-FU based chemotherapy group 1.0 *vs* 1.0). Gender, age at diagnosis, the WHO performance score, the number and extent of liver metastases were not significantly associated with the frequency of systemic inflammatory reaction.

## DISCUSSION

The rapid onset of spiking fever, fatigue, flu-like symptoms, and the increases in the serum CRP and inflammatory cytokine levels suggest that the patients treated with the combination of raltitrexed and carmofur or with single-agent raltitrexed developed a systemic inflammatory reaction a few days following chemotherapy infusions. Several lines of evidence suggest that the symptoms were not triggered by undiagnosed microbial infections, but that the major cause of symptoms was drug-related systemic inflammation. First, we failed to find evidence for the presence of microbial infection in the majority of patients despite extensive and repeated clinical examinations. Second, treatment with antibiotics appeared to have little effect on the clinical symptoms or the CRP levels. Third, the patients did not have neutropenia, an important risk factor for acute infection, on the days preceding the symptoms. The leukocyte nadir occurred on cycle days 10 to 14 after the raltitrexed infusion, whereas fever was usually present earlier, starting on the cycle days 2 to 4 days and resolving in all cases by the cycle day 9, whereas most of the verified infectious episodes developed after the day 7 of the cycle. Fourth, in most patients fever, fatigue, and the serum CRP elevations recurred following every chemotherapy cycle, which is not suggestive of infection-related fever, and the symptoms and CRP elevations were limited to patients who received raltitrexed. Taken together, although the presence of infection cannot be excluded with certainty as a cause of the symptoms, the present findings suggest that the symptoms and the related serum chemistry changes were caused by a treatment-related systemic inflammatory reaction.

The severity of the systemic inflammation, as defined by the systemic inflammatory composite score, appeared to increase with the increasing duration of raltitrexed treatment, and, in general, the combination of raltitrexed and carmofur caused more severe symptoms than raltitrexed alone. The symptoms and the concomitant elevations in the serum CRP and cytokine levels did not occur in patients treated with the 5-FU based regimens. Systemic inflammation that is not triggered by infection is apparently unusual in colorectal cancer patients treated with chemotherapy regimens consisting of 5-FU, oxaliplatin, or irinotecan (Andre *et al*, 1999a,b; Bleiberg and de Gramont, 1998; de Gramont *et al*, 1997; Saltz *et al*, 2000). However, similar symptoms have been observed in patients receiving bleomycin or cytarabine, which may induce inflammatory cytokine production (Chiche *et al*, 1993; Mishra *et al*, 2000; Sleijfer *et al*, 1998). The toxicities found in the present raltitrexed-based therapies resemble the effects linked to proinflammatory cytokines, such as IL-6 and TNFα. At present, the underlying mechanism(s) by which raltitrexed alone or in combination with carmofur triggers a systemic inflammatory reaction remain unknown.

Recurring fever, fatigue, and elevations of serum CRP could be inhibited with dexamethasone treatment in all patients tested. Hypothetically, a systemic inflammatory response triggered by raltitrexed might be beneficial by enhancing the immune defence against cancer. TNF-α and IL-6, which are pleiotropic cytokines, may have anti-tumour properties (Gluckman *et al*, 1997; May *et al*, 1988; Tanaka *et al*, 1997), and may be of benefit as chemotherapy agents in experimental cancer and even in the clinical treatment of colorectal cancer (de Vries *et al*, 1998, 1999; Lindner *et al*, 1999; Scheid *et al*, 1994; Tomita *et al*, 1998), and might act in synergy with conventional chemotherapeutics such as 5-FU (Oka *et al*, 1997; Watanabe *et al*, 1988). Increased anti-tumour activity associated with systemic inflammation might in part explain the as high as 50% response rate obtained by us with the combination of raltitrexed and carmofur in the treatment of metastatic colorectal cancer as the first-line therapy (Osterlund *et al*, 2001). However, the effect of dexamethasone on the treatment efficacy remains unknown and requires further study.

The severity of systemic inflammation is regulated at least partly by genetic factors (Louis *et al*, 1998). Systemic inflammation may lead to inflammation-mediated organ failure (Mira *et al*, 1999), and may also trigger a counter-reaction characterised by the development of immune suppression (Bone, 1996), which is associated with poor outcome in patients with sepsis (Volk *et al*, 1996). Although raltitrexed has a tolerable toxicity profile in general, the raltitrexed treatment-related mortality rate was recently found to be unexpectedly high (Anonymous, 1999; Maughan *et al*, 1999). Some of the patients with a fatal outcome had symptoms suggestive of systemic inflammation, and ultimately developed multiorgan failure. Many of the patients with severe toxicities did not have a proper dose reduction based on treatment-related neutropenia occurring in conjunction with diarrhoea or a deteriorating kidney function, which may have led to accumulation of toxicity (Anonymous, 1999; Maughan *et al*, 1999). Hence, although a systemic inflammatory reaction might have theoretical potential benefits by increasing tumour cell destruction, it might also have detrimental effects by eliciting toxicity, possibly leading to immune suppression and organ dysfunction.

We conclude that colorectal carcinoma patients treated with single-agent raltitrexed or the combination of raltitrexed and carmofur often develop fever, fatigue and elevation in the serum CRP and proinflammatory cytokine levels, which peak a few days after raltitrexed infusion and may recur after subsequent cycles. These features are suggestive for a drug-related systemic inflammation. The symptoms and laboratory findings may be difficult to differentiate from those caused by microbial infections. Corticosteroids may be effective in prevention of the symptoms, but they may mask underlying microbial infections, and their influence on the treatment efficacy is unknown.
